# Augmented Reality-Guided Extraction of Fully Impacted Lower Third Molars Based on Maxillofacial CBCT Scans

**DOI:** 10.3390/bioengineering11060625

**Published:** 2024-06-18

**Authors:** Marcus Rieder, Bernhard Remschmidt, Christina Gsaxner, Jan Gaessler, Michael Payer, Wolfgang Zemann, Juergen Wallner

**Affiliations:** 1 Division of Oral and Maxillofacial Surgery, Department of Dental Medicine and Oral Health, Medical University of Graz, 8036 Graz, Austria; 2 Institute of Computer Graphics and Vision, Graz University of Technology, 8010 Graz, Austria; 3 Division of Oral Surgery and Orthodontics, Department of Dental Medicine and Oral Health, Medical University of Graz, 8010 Graz, Austria

**Keywords:** augmented reality, cone beam computed tomography, molar, third, oral surgical procedure, surgery, computer-assisted, tooth extraction

## Abstract

(1) Background: This study aimed to integrate an augmented reality (AR) image-guided surgery (IGS) system, based on preoperative cone beam computed tomography (CBCT) scans, into clinical practice. (2) Methods: In preclinical and clinical surgical setups, an AR-guided visualization system based on Microsoft’s HoloLens 2 was assessed for complex lower third molar (LTM) extractions. In this study, the system’s potential intraoperative feasibility and usability is described first. Preparation and operating times for each procedure were measured, as well as the system’s usability, using the System Usability Scale (SUS). (3) Results: A total of six LTMs (*n* = 6) were analyzed, two extracted from human cadaver head specimens (*n* = 2) and four from clinical patients (*n* = 4). The average preparation time was 166 ± 44 s, while the operation time averaged 21 ± 5.9 min. The overall mean SUS score was 79.1 ± 9.3. When analyzed separately, the usability score categorized the AR-guidance system as “good” in clinical patients and “best imaginable” in human cadaver head procedures. (4) Conclusions: This translational study analyzed the first successful and functionally stable application of the HoloLens technology for complex LTM extraction in clinical patients. Further research is needed to refine the technology’s integration into clinical practice to improve patient outcomes.

## 1. Introduction

The extraction of impacted lower third molars (LTMs) constitutes one of the most prevalent procedures in the field of oral and maxillofacial surgery [1,2]. The indications for LTM removal include therapeutic reasons, such as acute or chronic pericoronitis, cyst formation, or non-restorable caries lesions, as well as prophylactic considerations [3]. Concerning prophylactic extraction, the “hygienic cleansability” of the LTM constitutes the most important factor in terms of the prevention of pathological conditions [4]. This routinely performed procedure is generally associated with a low incidence of intra- and postoperative complications [5,6]. In this context, an approximate incidence rate of around 5% infection, 1.5% postoperative bleeding, and 6% temporary and 2% permanent sensory disturbances are documented [7,8]. However, although only a low complication incidence is known regarding LTM extraction, various biomaterials can additionally be used to enhance wound healing. Deeply impacted and/or mispositioned LTMs close to the inferior alveolar nerve (IAN) can lead to challenging and time-consuming surgical procedures, which can harm the patient [9,10]. Such procedures risk prolonging the postoperative wound healing due to increased damage of the mandibular bone, damage to neighboring teeth due to the expanded surgical access, or temporary or permanent neurosensory deficiency affecting the lower ipsilateral side of the lip, chin, buccal gingivae, and teeth [11].

To avoid damage to such critical structures and minimize the risk of postoperative complications, modern medical imaging techniques, including computed tomography (CT), cone beam computed tomography (CBCT), and magnetic resonance imaging (MRI), are often routinely used as primary information resources aiding the surgeon to better assess the individual patient situation. However, independent of the used imaging technique and its associated accuracy, the visualized image data are displayed spatially and temporally detached from the patient and separated from the operating field on a screen. This dichotomy results in a cognitive challenge for the surgeon; the physician must continually mentally correlate information between 2D imaging data displayed on a screen and the three-dimensional anatomy of the patient [12]. This is still the case when additional computer-based software solutions are used to support the operative procedure, such as image-guided surgery (IGS) and others. Moreover, the implementation of complex software systems, such as IGS, usually involves additional substantial expense and effort, including patient registration and calibration procedures, which may not be justified for the specific context of LTM extraction [13,14]. Consequently, IGS is only accessible in clinical centers with the necessary financial resources.

In this context, augmented reality (AR) emerges as a transformative solution capable of addressing these challenges by seamlessly integrating radiological imaging data in three dimensions directly into the current clinical scenario and the patient [15,16]. The application of AR in oral and maxillofacial contexts is presently being investigated across a wide field of various domains. Among others, these domains encompass: implantology, orthognathic surgery, reconstructive surgery, oral surgery, orthodontics, and endodontics, and may also be integrated into training and teaching procedures [17,18,19,20,21,22,23,24,25,26].

In this context, the HoloLens 2 (HL) from Microsoft (Microsoft Corp., Redmond, WA, USA) stands out as a prominent AR hardware device in the healthcare domain, with particular significance in dental procedures involving the craniofacial region [27]. This see-through head-mounted display allows users to interact with virtual objects in their physical environment, offering a superior alternative to traditional IGS systems. Unlike conventional setups, the HL provides surgeons with real 3D visualization of patient-specific data without the need to shift attention between a monitor and the patient. Its cost-effectiveness and slim form factor further enhance its appeal. Notably, Gsaxner et al. have introduced a practical IGS system based on the HL, offering advantages such as simplified assembly and the elimination of external hardware or infrastructure requirements [28,29,30]. This innovative system makes use of routinely acquired pre-interventional scans to extract data about a patient’s skin surface. Through the adeptness of the HL hardware, it achieves automatic and markerless registration between the extracted skin model and the physical patient. This development showcases the HL’s potential for enhancing dental procedures with streamlined and efficient AR image guidance. Unlike other dental guidance systems, it potentially sidesteps the need for any external navigation and/or bulky markers, indicating a significant step forward in leveraging see-through AR in dental and maxillofacial surgery [17,31]. Despite this progress in 3D visualization, the full potential of the HL in dental and maxillofacial surgery remains largely unexplored and is still undergoing scientific investigation.

Therefore, the objective of this translational study is to integrate an AR guidance system based on CBCT scans into clinical practice, specifically to assess the system’s usage in AR-guided complex impacted LTM extractions. The null hypothesis of this study is that the use of an AR-guided system for fully impacted third molar removal is not sufficiently feasible in human cadaver heads nor, therefore, in clinical patients.

## 2. Materials and Methods

In this study, both preclinical and clinical surgical setups were employed to accordingly assess and implement the AR technology regarding impacted LTM extractions. In a preclinical setup, fully impacted LTM extraction was conducted using human cadaver head specimens as a first step. The clinical setup, as a second step, included the extraction of fully impacted LTMs in clinical patients. The primary objective of these procedures was to evaluate the potential advantages offered by the AR guidance system. Preceding each surgical intervention, a CBCT scan was performed, and the anatomical structures of interest (impacted LTM, mandible, maxilla, IAN channel) were segmented preoperatively for a subsequent intraoperative AR visualization. The three-dimensional data generated from these scans were precisely aligned before the initiation of the surgical procedure.

### 2.1. Sample Collection

For the preclinical setup, cadaveric head specimens were procured from the Division of Macroscopic and Clinical Anatomy at the Medical University of Graz, Austria. The preservation method, based on Thiel’s technique, was meticulously employed to maintain the high-quality standard of all the specimens [32]. Approval for the utilization of post-mortem tissues was obtained in accordance with the Styrian Death and Funeral Act of 2010, following a thorough institutional review. The handling of all the specimens adhered strictly to the guidelines outlined by the donation program of the division. For the clinical setup, the patients’ informed consent was given by all the participants in the clinical investigation. All the patients and specimens included were treated at the University Clinic of Dental Medicine and Oral Health at the Medical University of Graz, Austria between November 2023 and March 2024.

### 2.2. Human Head Cadaver Specimens

**Figure 1:**
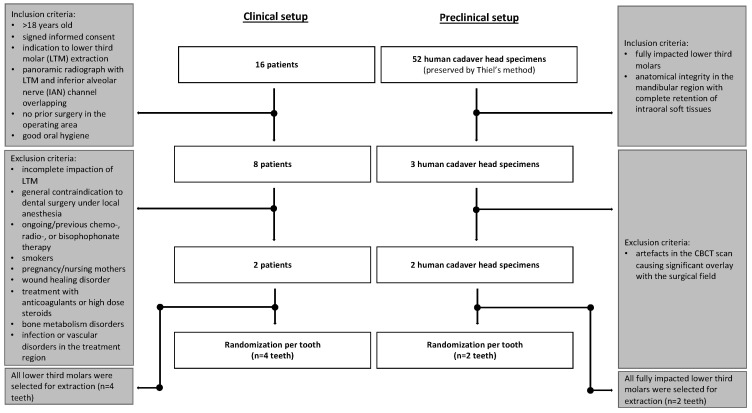
The flowchart illustrates the enrollment and allocation of the clinical (**left side**) and the preclinical trial (**right side**) of the present study.

**Figure 2:**
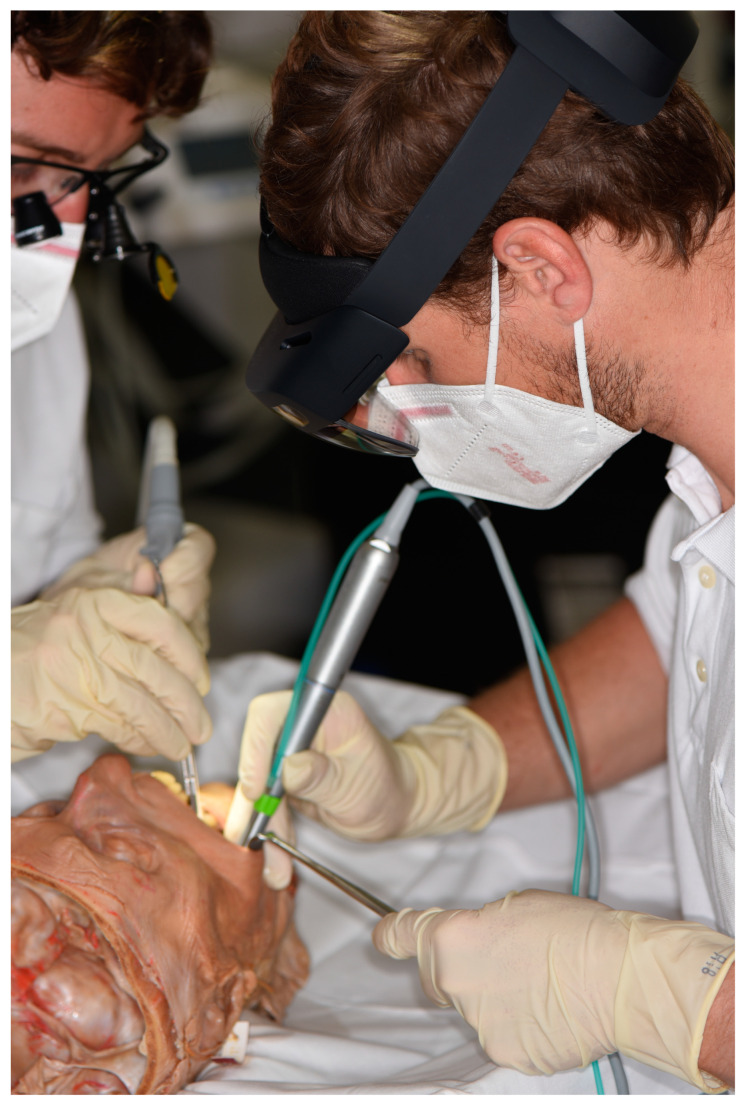
Pre-clinical setting: Intraoperative view of extraction of the fully impacted right lower third molar on a human cadaver head specimen. The surgeon, equipped with a HoloLens, is performing the osteotomy, while the assistant provides sufficient lighting using magnifying loupes.

Fifty-two human cadaveric head specimens (*n* = 52) underwent a preliminary CBCT scan using the Orthophos CBCT scanner (Dentsply Sirona, Bensheim, Germany), with parameters set at 96 kV, 5.6 mA, an exposure time of 9.335 s, a field of view (FOV) measuring 23 × 27.5 mm, a voxel size of 0.400 mm, and a slice thickness of 1 mm. This screening allowed for the assessment of the specimen’s eligibility. To enable an objective and comparable sample, only fully impacted LTMs were considered for inclusion in this study. Furthermore, an additional inclusion criterion for participation was the preservation of anatomical integrity in the mandibular region, coupled with the complete retention of intraoral soft tissues. After applying the aforementioned inclusion criteria, two human cadaver head specimens (*n* = 2), each with an impacted LTM (*n* = 2), were included in the study (Figure 1). Figure 2 shows an intraoperative scene on a human head cadaver specimen.

### 2.3. Clinical Patients

#### 2.3.1. Eligibility/Inclusion Criteria

This study included individuals: (1) over 18 years of age; (2) with signed informed consent; (3) presenting with a medical indication to extract at least one fully impacted LTM; (4) with a 2D panoramic radiograph indicating contact or overlap of the LTM and the mandibular canal; (5) missing prior experience of surgical procedures in the operating area (e.g., bone harvesting, etc.); (6) able to achieve good postoperative oral hygiene.

#### 2.3.2. Exclusion Criteria

The exclusion criteria comprised: (1) incomplete impaction of the LTM; (2) general contraindications to dental surgery under local anesthesia (e.g., severe systemic diseases, tumors, severe cardiovascular diseases, uncontrolled diabetes mellitus, etc.); (3) ongoing or previous chemotherapy, radiotherapy, or bisphosphonate therapy; (4) self-reported smokers; (5) pregnancy and nursing mothers; (6) disorders or treatments that impair wound healing; (7) long-term treatment with high-dose steroids or anticoagulants; (8) bone metabolism disorders; (9) infections or vascular disorders in the treatment region.

**Figure 3:**
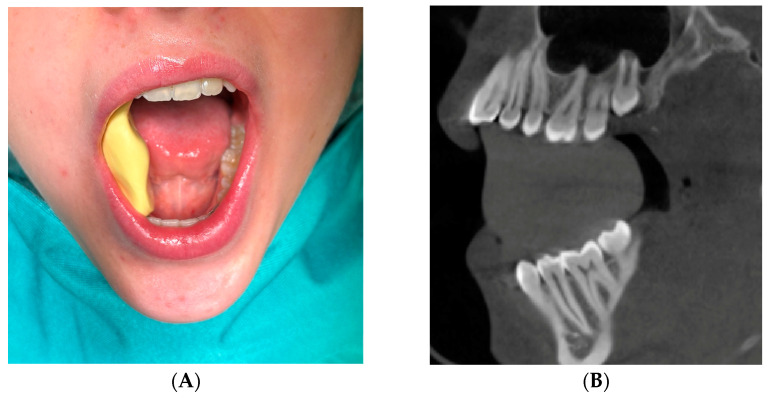
(**A**) Intraoperative view of the patient with the bite block in place before surgery; (**B**) Cone beam computed tomography image in the sagittal plane displaying the bite block that stabilizes the mandible in a standardized position, eliminating visualization errors due to jaw movement.

Patients meeting the inclusion criteria who came for consultations on LTM extractions from September 2024 to November 2024 were invited to participate in this study. After identifying two eligible participants, standard-dose CBCT scans of the clinical patients were performed using the Planmeca ProMax 3D Max system (Planmeca, Helsinki, Finland). The field of view for the scans was set to ensure coverage of at least one complete dental arch. The scans utilized a 200 mm voxel size, with parameters set at 96 kV, 5.6–9.0 mA, and an exposure time of 12 s. This imaging protocol was selected to facilitate thorough and detailed visualization for the subsequent implementation of head-mounted visual AR guidance technology in the study. To address the challenge of unreliable registration of structures in the lower jaw, CBCT scans were conducted using a customized bite block (Optosil comfort putty, Kulzer, Mitsui Chemicals Group, Tokyo, Japan). This bite block was consistently employed throughout the entire surgical procedure (Figure 3).

### 2.4. Augmented Reality System

The implemented AR IGS, leveraging the second version of the HL, represents a cutting-edge technology designed to enhance the surgical visualization experience. The system’s core feature is its self-localization algorithm embedded in the HL, allowing for precise mapping of the user’s surroundings and localizing the device within. This not only enables the intuitive placement of virtual content but also contributes to a highly immersive experience for users.

The distinct facial features and relative rigidity of facial structures are exploited for patient registration. Using video frames from the HL camera and depth maps from the time-of-flight depth sensor, a deep learning-based, single-shot, multi-box detector identifies and tracks a bounding box around the patient’s face in real time [33]. This bounding box is then mapped to the depth frame, reconstructing a point cloud representation of the patient’s face through inverse perspective transformation. A two-stage point-based registration algorithm aligns the point cloud with the 3D model of the patient’s skin surface obtained from the pre-interventional CBCT data. This involves an initial fast global registration followed by refinement using an iterative closest-point approach, ensuring accurate alignment with the actual patient anatomy [34,35]. The AR system is controlled through a virtual user interface (UI) that enables seamless interaction. Fully automatic patient registration is initiated by examining the patient, with continuous updates to the position and orientation of virtual content to accommodate patient movement and enhance alignment precision. Manual refinement of the registration is also possible, especially when accounting for perceptual misalignments due to individual anatomy or soft tissue deformations. The UI, conveniently locked to the user’s left hand, provides options to switch between different registration modes, select anatomical structures for visualization, and adjust parameters related to content positioning and visualization. This flexible interface enhances user control and allows for a customized and efficient AR experience. Closing the left hand locks the UI in its current position in the room, providing stability and ease of use during interventions. In summary, the AR system combines innovative technological features and user-centric design elements to offer a sophisticated and adaptable platform for enhancing surgical visualization and precision [30].

### 2.5. Randomization and Training

Following the selection of patients and human cadaver head specimens, the allocation of each LTM to one of four experienced surgeons was performed using digital randomization software (Version 2.1.0, Institute for Medical Informatics, Statistics and Documentation, Medical University of Graz, Graz, Austria, and a randomizer for clinical trials: www.randomizer.at accessed on 27 November 2023; [36]) by an individual not involved in the treatment procedure.

All four surgeons underwent a comprehensive orientation to the HL, along with an in-depth exploration of the virtual UI. This orientation facilitated their familiarization with various visualization modes, serving as a preparatory training regimen. Multiple training sessions were conducted in advance of the present study, affording the surgeons sufficient time to acclimate to the system. This prolonged exposure contributed to the development of expertise and confidence in navigating the virtual environment. Consequently, the training intervention yielded a diminished learning curve, mitigated performance bias, and optimized efficiency during the subsequent evaluation task.

Moreover, the early training sessions allowed for the identification and resolution of minor system-related issues, ensuring a seamless and unencumbered execution of the study. This proactive approach not only bolstered the surgeons’ readiness but also fostered an environment conducive to reliable data collection during the evaluation.

### 2.6. Segmentation and Preparation

**Figure 4:**
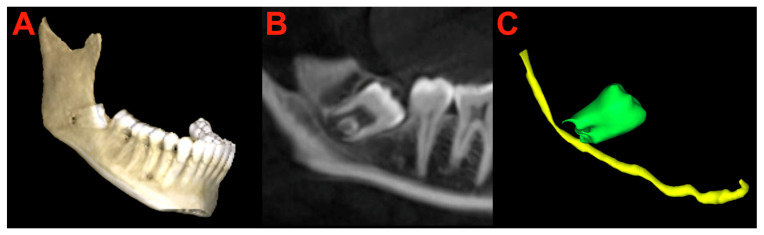
(**A**) Preoperative automatic rendering of the lower jaw depicting the fully impacted right lower third molar (LTM). (**B**) Sagittal plane of the cone beam computed tomography illustrating the relationship between the roots of the right LTM and the inferior alveolar nerve (IAN) channel. (**C**) Preoperative rendering of the right lower third molar using 3D Slicer software. A three-dimensional model of the right IAN (yellow) and LTM (green) is shown.

Preoperatively, the complete LTM and the IAN channel were individually marked in the preoperative CBCT scan with the assistance of 3D Slicer software (Version 5.4.0, Slicer Community, USA; available at: https://www.slicer.org, accessed on 2 November 2023) (Figure 4). Additionally, the entirety of the facial skeleton and skin surface underwent segmentation for subsequent visualization in AR. This involved automatic segmentation, specifically through thresholding, followed by careful manual refinement.

Automatic patient registration aligned the CBCT imaging with the patient/specimen. In certain cases, manual adjustments were made to the registration. Following this, anatomical structures and associated visualization parameters were tailored to the specific procedure’s requirements. The duration of these preparatory steps, recorded as “preparation time”, also encompassed visual documentation.

### 2.7. Surgery

All the surgical procedures followed a standardized protocol of the University Clinic of Dental Medicine and Oral Health, Medical University of Graz, Austria [37]. Initial procedures involved local anesthesia administered via a nerve block targeting the IAN and the lingual nerve (LN). Supplementary depots were strategically administered along the ascending mandible to anesthetize the buccal nerve, and submucosal depots were executed in the buccal region corresponding to teeth 37 and 47.

Surgical access was gained through an incision made at the marginal gingiva of teeth 46 to 47, with simultaneous detachment of the dental papilla. The incision was extended from the distobuccal side of tooth 47 on the ascending mandible into the vestibule. Subsequently, a full-thickness envelope flap was meticulously raised using a freer. The retractor was employed to hold off the buccal portion of the flap, while a curved periosteal elevator was cautiously inserted subperiosteally on the lingual side to ensure the preservation of the LN.

**Figure 5:**
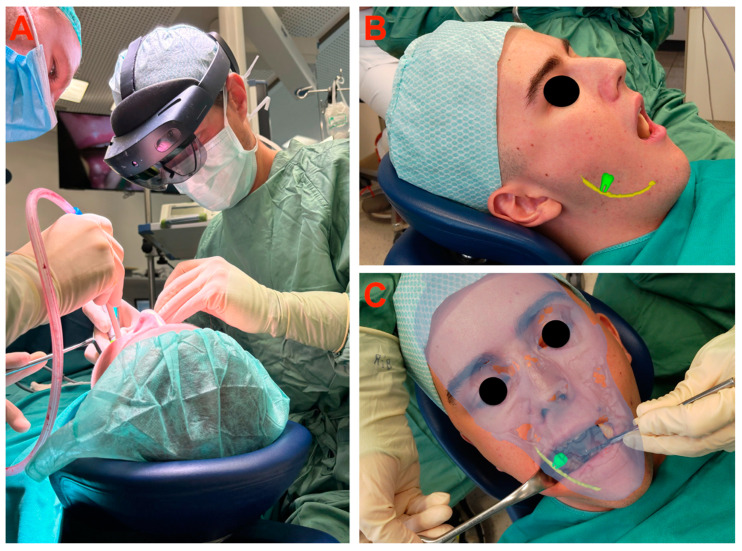
Clinical setting: (**A**) Intraoperative scene depicting the surgeon equipped with the HoloLens 2. (**B**) Real-time preoperative view captured with the HoloLens 2 before extraction of the lower third molar. Augmented reality superimposition of the lower third molar (green) and inferior alveolar nerve (yellow) over the patient. (**C**) Preoperative view with the addition of the bony skull structure (blue) through the user interface.

**Figure 6:**
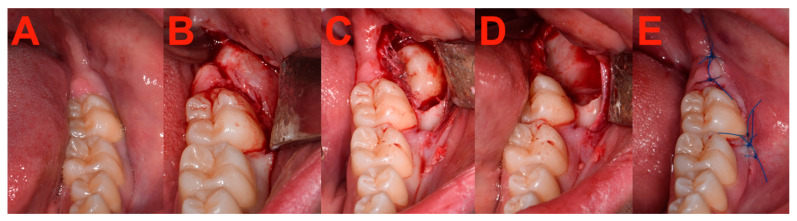
Intraoperative scene showcasing steps of extraction of the fully impacted lower third molar (LTM). (**A**) Preoperative intraoral situation depicting a fully impacted left LTM (**B**) Intraoperative view after raising the full-thickness envelope flap. (**C**) Visible LTM after osteotomy. (**D**) Empty alveolar socket after removal of the LTM. (**E**) Intraoral view after wound closure with non-absorbable sutures.

The osteotomy procedure was conducted using a rose bur until the complete tooth crown was exposed. If necessary, the tooth was fragmented into pieces using a Lindemann bur. The removal of the tooth or its individual pieces was carried out either by a lever or using surgical clamps. Closure of the wound was achieved through the application of non-absorbable sutures. The intraoperative setup is shown in Figure 5 and Figure 6.

### 2.8. Measurements

For each tooth subjected to LTM extraction, careful documentation of both preparation and operating times was undertaken. The operating time was divided into two phases, delineated as the duration from incision to complete LTM extraction and the subsequent wound closure period. Precise time measurements were conducted utilizing a calibrated stopwatch. Preparation time, measured in seconds, and operating time, rounded up to minutes, were systematically recorded. Logged times were later compared to the corresponding metrics obtained from 12 fully impacted previously conducted LTM extractions by the same four experienced surgeons (3 LTM each), which served as a benchmark for the AR-assisted surgeries.

To assess the efficacy/usability of the AR guidance system based on CBCT scans in the context of LTM extractions, the well-established System Usability Scale (SUS) was employed. The SUS, recognized as a validated and standardized questionnaire, gauges the ease of use across diverse products and services, including applications and hardware. Comprising 10 items rated on a 5-point Likert scale [38], the SUS yields scores that range from 0 to 100. It is imperative to note that these values are not presented as percentages and should be interpreted exclusively based on their percentile ranking [39].

### 2.9. Statistical Analysis

The statistical analyses involved descriptive calculations, with the data presented as the mean ± standard deviation (SD) or median and interquartile range (IQR). All the statistical analyses utilized the Python package “pingouin”. To enhance data visualization, tables and boxplots were employed. Prior to usage, all the specimens/patients were anonymized. Data collection was conducted through a case report form, adhering to legal regulations governing the collection, transfer, and storage of human cadaver specimens’/patients’ image data within the study.

## 3. Results

A total of six LTMs were analyzed in this study (*n* = 6), comprising two from human cadaver head specimens and four from clinical patients. All included LTMs were radiologically confirmed as fully impacted, with no evidence of radiolucency surrounding the tooth in CBCT scans, and were in close association to the IAN. Extractions of the LTMs from human cadaver head specimens were performed by two experienced surgeons (BR, JW), while those from living patients were carried out by four experienced surgeons (BR, JW, MR, MP). There were no observed injuries to critical anatomical structures, including adjacent roots, the IAN, or surrounding soft tissues. Furthermore, no postoperative complications such as neurosensory deficits, wound healing disorders, postoperative bleeding, or infections were documented. All the case characteristics and SUS scores are shown in Table 1.

### 3.1. Measurements

The preparation times ranged from 118 to 240 s, with a mean of 166 ± 44 s. The preparation time included automatic patient registration as well as manual adjustments (if necessary). The mean operating time was 21.3 ± 5.9 min, equivalent to the time from incision to the start of the suture. Median suturing time was 245.5 ± 57 s. It is worth noting that the operating times on the human head cadaver specimens were shorter compared to the clinical surgery on patients.

### 3.2. System Usability

The effectiveness of the AR-guided LTM extractions, utilizing CBCT scans, was assessed using the established SUS score. Postoperatively, each surgeon directly completed the questionnaire in order to evaluate their performance and the usability of the head-mounted HL-based augmented reality system. The mean SUS score was 79.1 ± 9.3, and scores ranged from 70 to 92.5. According to Brooke’s method of score interpretation, these values both indicate a high usability level [38]. Furthermore, the scores indicate a usability level described as “good” for patients and “best imaginable” for procedures on human head cadavers.

## 4. Discussion

The objective of the present study was to assess the usability of an AR IGS based on CBCT scans in a clinical and preclinical setup to support fully impacted LTM removal. In this context, six complex LTM extractions were performed using the second generation of the Microsoft HL. Two (*n* = 2) of these LTM extractions were performed on human cadaveric head specimens and four (*n* = 4) on patients.

The extraction of impacted LTMs is a recurrently performed surgical intervention and represents the most common operation in the field of oral and maxillofacial surgery [1]. Although the surgical removal of LTMs is routinely undertaken mostly in young and healthy patients as a preventative measure, the surgical procedure is also undertaken in response to the presentation of acute symptoms that may necessitate immediate surgical attention [8,40]. Even when performed routinely, this surgical procedure carries inherent risks and side effects. A primary risk factor, especially in fully deep-impacted LTM, is damage to the associated nerves. Damage to the IAN, in particular, can result in transient or permanent neurosensory deficits. The damage to these structures represents a significant complication resulting in sensory impairments of the lower lip and/or tongue and has a high potential to affect the patient’s quality of life adversely [41,42].

To reduce the surgical risk to these sensitive structures, computer-based surgical navigation techniques were developed. Such navigation techniques can roughly be divided into two types: static navigation (SN) and dynamic navigation (DN). Despite their innovative approaches, these techniques face a variety of obstacles such as high costs, licensing restrictions, bulky setups, and increased mental workload due to the need to simultaneously monitor both screen and patient, leading to decreased hand–eye coordination [12,43]. In this context, AR may overcome numerous limitations associated with SN and DN by effortlessly merging three-dimensional radiological imaging data into the present clinical situation and directly overlaying it on the patient [15,16]. In the literature, the HL has already been investigated regarding the successful use for several AR indications in oral and maxillofacial surgery [43,44,45,46,47].

However, limited literature exists that addresses the navigation-based treatment of LTM, with the majority of studies concerning DN systems. Zhang et al. demonstrated that using DN within an LTM coronectomy significantly improves precision by completely removing the enamel, leading to greater surgical success and better patient recovery outcomes [48]. A coronectomy, in which the crown is cut and removed while the root is preserved, is described to reduce the risk of injury to the IAN compared to conventional extraction methods [49]. Emery et al. investigated DN for the extraction of complex LTMs and demonstrated its advantages, including improved visualization of critical structures like the IAN, increased precision in osteotomy, reduced bone removal, decreased need for extensive surgical access, and reduced surgical time. However, potential drawbacks included high costs, additional time required for preoperative planning, and a learning curve for new users [50]. Pellegrino et al. conducted a study highlighting the advantages of DN in the extraction of impacted LTMs. These advantages include avoiding soft tissue detachment, minimizing bone loss, preserving nearby critical anatomical structures, and reducing intraoperative bleeding through precise multi-sectioning of the tooth. However, the application of DN was limited to a case series involving three patients in which the LTMs were only partially erupted [51]. Furthermore, it should be noted that several authors have successfully used DN to treat iatrogenically displaced wisdom teeth or their roots in soft tissues, as well as iatrogenically placed foreign bodies in the mandibular bone. All have described DN as providing significant advantages for the surgical procedures [52,53,54,55].

Instead of DN, the HL was utilized in the present study to support surgeons during the removal of fully impacted LTMs. As described, both the impacted LTM and the IAN were preoperatively segmented and superimposed in the surgeon’s field of view during the operation. This was intended to facilitate a clearer understanding of the sometimes complex position of the tooth within the bone, as well as its relationship to the IAN while operating. Usage of the HL offers significant opportunities to circumvent many limitations of conventional navigation systems previously discussed. Manageable costs and a short setup time, which was 118 to 240 s in the present study, may reduce barriers to integrating this system into daily use within both scientific settings and clinical practices. In addition, the statistics demonstrated that the operating times associated with the use of the HL in the clinical setup (mean 24.8 ± 3.4 min) were comparable to other clinical operating times where LTM removal was done by free hand without navigational support. Therefore, a total of twelve procedures performed by the same surgeons without the HL were collected as a reference value (mean 21.2 ± 5.6 min) for comparison. The similarity of the mean values indicates that the use of AR technology does not significantly prolong operation times. Regarding the additional effort of the system’s preparation (e.g., data preparation, segmentation), various authors suggest that this burden could be significantly reduced with ongoing advancements in automatic segmentation [56,57,58,59].

Nevertheless, it should be noted that the operation times for LTM removal from human cadaver head specimens in the preclinical setup were shorter than in the clinical setup. This difference can be attributed to various time-consuming patient-related factors occurring only clinically, such as smaller surgical access due to reduced mouth opening compared to cadaver heads, and/or additional handling, such as suction and others needed because of bleeding and wound exudation. Furthermore, the prolonged intraoperative pain management encountered during clinical procedures, as well as the movement of the patient’s head that necessitates a new system calibration, must be taken into consideration as an additional time-consuming factor clinically.

In terms of the system’s usability, the current study judged the category “best imaginable” for the extraction of the LTMs on the human cadaver head specimens. The SUS score recorded during the use of the HL on clinical patients was categorized as “good”, which is the third best level in the seven-point rating scale (i.e., worst imaginable (1) to best imaginable (7)). The discrepancy between the clinical and preclinical usability scores can be attributed to the same aforementioned factors, which may have also influenced the time differences. Similar results regarding the SUS score were reported by Remschmidt et al. for the use of the HL during apicoectomies (i.e., mean 80.4 ± 6.8). However, the study of Remschmidt et al. was conducted exclusively on human cadaver head specimens rather than clinical patients.

This translational study analyzed the first successful and functional stable application of the HL technology for complex LTM removal in clinical patients. Although, there was some latency observed in the streaming-based data transfer (send/return data between the HL and computer hardware), the use of the AR HL system for LTM removal worked in the clinical and preclinical settings. The successful removal of fully impacted third molars using an AR-guided system in both human cadaver heads and clinical patients, supported by the usability scores and the history of comparable operation times, disproves the null hypothesis of this study.

Despite evidence demonstrating the usability of this novel technology, certain shortcomings in this study require further research. The study’s sample size is insufficient to provide adequate evidence to generalize the research results. However, given the recent introduction of the system into a clinical setting, the aim at this stage was to demonstrate its feasibility. Although the potential misalignment of the mandible’s structures due to the lower jaw’s mobility was addressed with a customized bite block (which ensured a predefined position of the lower jaw throughout the entire surgery and the CBCT scan) a non-rigid registration could enable the use of three-dimensional image data without such devices. Furthermore, the field of operation is often confined to millimeters, and AR overlays can be accurately performed after meticulous calibration under static conditions. However, ensuring such ideal scenarios throughout the surgery becomes inherently challenging. The experienced latency caused by the streaming-based approach should primarily be improved upon by optimizing the hardware and technology. It should further be noted that quantifiable parameters regarding the usability and assessment of such technologies are hard to find. To overcome this problem, additional studies are necessary to further evaluate the system and associated patient-related outcome variables, as well as objectively quantifiable parameters to more clearly elucidate the possible advantages of this technology.

## 5. Conclusions

This is the first structured clinical analysis of AR-guided extractions of fully impacted LTMs using the HL and CBCT scans. The system’s usability score in a clinical setting can be described as “good” for fully impacted LTM extraction. During the operative procedure, a functional stable application of the system was possible in the clinical patients. In strong contrast to other navigation systems, the employed AR system offers the substantial advantage of transferring critical anatomical structures directly into the operation field and the surgeon’s view. This visualization technology may help to reduce risks associated with the operative procedure and increase the intraoperative imagining of the tooth position and its surrounding anatomical structures, which are prone to be harmed. With technological advancements, AR systems are poised to revolutionize dentistry, providing new methods to enhance patient care. Additional research is essential to overcome existing limitations and further integrate this technology into daily practice.

## Figures and Tables

**Table 1:** Detailed Case Characteristics and System Usability Scale Score. HHCST = human head cadaver specimen tooth, PT = patient tooth.

Case ID	Tooth Region	Preparation Time (min:s)	Operation Time (min)	Suture Time (min:s)	SUS Score (System Usability Scale)
HHCST 1	48	04:00	14	02:55	87.5
HHCST 2	48	03:00	15	03:15	92.5
PT 1	38	02:00	28	05:35	70
PT 2	48	02:50	25	04:31	70
PT 3	48	01:58	20	04:22	75
PT 4	38	02:50	26	03:55	80

## Data Availability

The original contributions presented in the study are included in the article, further inquiries can be directed to the corresponding author.

## Abbreviations

AR	augmented reality
CBCT	cone beam computed tomography
CT	computed tomography
DN	dynamic navigation
FOV	field of view
HL	HoloLens 2
IAN	inferior alveolar nerve
IGS	image-guided surgery
IQR	interquartile range
LN	lingual nerve
LTM	lower third molars
MRI	magnetic resonance imaging
SD	standard deviation
SN	static navigation
SUS	system usability scale
UI	user interface
